# Neuroticism vulnerability factors of anxiety symptoms in adolescents and early adults: an analysis using the bi-factor model and multi-wave longitudinal model

**DOI:** 10.7717/peerj.11379

**Published:** 2021-06-22

**Authors:** Yini He, Ang Li, Kaixin Li, Jing Xiao

**Affiliations:** 1 Key Laboratory for NeuroInformation of Ministry of Education, School of Life Science and Technology, University of Electronic Science and Technology of China, Chengdu, China; 2 State Key Laboratory of Brain and Cognitive Science, Institute of Biophysics, Chinese Academy of Sciences, Beijing, China; 3 School of Mechanical and Power Engineering, Harbin University of Science and Technology, Harbin, China; 4 Beijing Key Laboratory of Learning and Cognition, School of Psychology, Capital Normal University, Beijing, China

**Keywords:** Neuroticism, Anxiety symptom, Bi-factor model, Multi-wave longitudinal model

## Abstract

**Background:**

Neuroticism and stress are important vulnerability factors in the development and outcome of anxiety symptoms. However, as neuroticism is a heterogeneity trait, it is still unclear how different neuroticism factors contribute to anxiety symptoms independently or in conjunction with stress. Thus, different factors of neuroticism were extracted in the present longitudinal study using the bi-factor model. The prediction effect of these different factors on anxiety symptoms and their combined effects with stress in both adolescent and adult samples were examined.

**Method:**

Participants (592 adolescents and 638 young adults) in Hunan China were included. In the initial assessment in our longitudinal study, participants were asked to complete measurements that assessed neuroticism, stress, and anxiety symptoms. Next, a monthly assessment of stress and anxiety symptoms was completed for the subsequent 6 months. The bi-factor model was used to extract different factors of neuroticism. The hierarchical linear model was used to analyze longitudinal multi-wave data.

**Result:**

Several model fit indices were used to evaluate the bi-factor model fit for neuroticism (adolescent: Tucker-Lewis index (TLI) = 0.957, comparative fit index (CFI) = 0.973, RMSEA = 0.040, Chi-Square = 80.471; early adults: TLI = 0.957, CFI = 0.973, RMSEA = 0.042, Chi-Square = 88.465). The results of hierarchical linear modeling analyses indicated that the general factor of neuroticism possessed a predictive effect on anxiety symptoms (adolescents: F = 36.77, *p* < 0.0001, early adults: F = 30.44, *p* < 0.0001); The negative effect factor only had the prediction effect on anxiety symptoms in early adults (adolescents: F = 0.65, *p* > 0.05; early adults: F = 4.84, *p* < 0.05); No prediction of self-reproach factor was found on anxiety symptoms (adolescents: F = 3.79, *p* > 0.05; early adults: F = 0.02, *p* > 0.05); the interactive effects of the general factor and stress on anxiety symptoms were only found in early adulthood (adolescents: F = 0.13, *p* > 0.05; early adults: F = 11.55, *p* < 0.01).

**Conclusion:**

Our results suggested that the bi-factor model achieved a satisfactory fit for neuroticism measurement and supported that the anxiety symptoms were induced by the main effects of the general factor in both age samples and the negative factor only in adults. The general factor of neuroticism, but not the negative factor could make an additive effect for anxiety symptoms in face of stress, which meant that the homogeneity of neuroticism played a more significant role in further anxiety symptoms than heterogeneity when coping with stress.

## Introduction

Anxiety is one of the most common mental disorders worldwide, and numerous epidemiological studies have shown that the incidence rate of this disease is increasing rapidly in teenagers and young adults (Beesdo, Knappe & Pine, 2009; World Health Organization, 2017). The symptoms of anxiety disorder are manifested as painful emotional experiences that are not commensurate with the situation, such as psychomotor restlessness (e.g., fell restlessness) or vegetative nerve dysfunction accompanied by physical discomfort (e.g., dyspnea), which not only bring problems to the patients’ families, but also greatly affect their social function, life satisfaction, and so on (Andrews et al., 2010; Oltmanns et al., 2018). For years, studies have sought to identify the susceptibility factors of anxiety symptoms (Keogh & Reidy, 2000; Wardenaar et al., 2010).

In studies concerning the identification of risks that lead to the development of anxiety symptoms, the diathesis stress model is one of the most important and common methods employed (Auerbach et al., 2007; Gazelle & Rubin, 2019; Kendler, 2020). The model assumes that the onset of mental illness results from the risk features (diathesis) of the individuals exposed to a certain intensity of stressor (stress) (Gazelle & Rubin, 2019). In self-diathesis, neuroticism is considered as one of the most significant risk factors that lead to anxiety (Vittengl, 2017; Williams et al., 2020). Based on the five-factor model of personality, neuroticism is defined as the tendency to experience negative emotions and emotional stability (Cho, Kim & Park, 2017; Ueda et al., 2018). Previous researchers have found evidence of overlapping genetic determinants between neuroticism and some affective disorders (such as anxiety and depression) and indicated that personality traits represent at least part of what is inherited in anxiety disorders (Bienvenu & Stein, 2003; Weinstock & Whisman, 2006). However, it remains unknown how neuroticism affects anxiety symptoms.

This question is likely for the following reasons. First, although longitudinal researches can reflect causal relationships, there are fewer longitudinal studies on the relationship between neuroticism and anxiety available than cross-sectional studies (Bienvenu & Stein, 2003). Second, such past studies mainly focused on adults, and relatively few included different age groups. However, anxiety symptoms at a young age are probably a sign of disease, and the best time for intervention may be missed if they are not prevented or controlled in time (Cartwright-Hatton, McNicol & Doubleday, 2006; Neil & Christensen, 2009; Sawyer et al., 2012). Moreover, the brain is relatively plastic during adolescence, and so, adolescence is a particularly important period for prevention and intervention (Giovanelli, Ozer & Dahl, 2020). With this in mind, separate verifications in the adolescent and early adult samples will aid the identification of anxiety risk factors at different ages. Third, some evidence has emerged to challenge the traditional view that neuroticism is viewed as a homogeneous and negative trait in recent years (Chapman, Roberts & Duberstein, 2011). For instance, a recent study has showed that individuals with high level of neuroticism can be more positively creative when the anxiety state is aroused (Leung et al., 2014). And other studies have also suggested that artists with low neuroticism trait are actually less creative in comparison with highly neurotic individuals (Booker, Fearn & Francis, 2012; Feist, 1998). What is even more important is that traditional sum-score method, which is commonly used in neuroticism assessment, regards neuroticism as a homogeneous trait and ignores individual difference and heterogeneity within neuroticism (Hill et al., 2020). Recent studies found that the latent variable model can well reflect the heterogeneity within personality traits and extract different facets of factors that have better predictive validity for the structure of personality than the results from the traditional sum-score method. For example, studies used the bi-factor model (one of commonly used latent variable model) to extract a general factor and two special factors of neuroticism, and then reported that these factors showed different effects on mortality and intelligence (Gale et al., 2017; Hill et al., 2020). Their results explain in more detail when and how neuroticism affects behavior or other traits. These experiences have suggested that measuring the different factors of neuroticism based on the bi-factor model will help us to better understand how neuroticism affects anxiety symptoms.

In addition, the diathesis stress model of anxiety symptoms should emphasize the interaction between neuroticism and stress. Some studies have shown that both neuroticism and stress can influence anxiety, but recently researchers start to emphasize whether the effects of diathesis (or vulnerability factor) and stress could be interactive or additive (Mineka et al., 2020). Specifically, the additive diathesis-stress model hypothesizes that neuroticism and stress have direct effects on anxiety, but without a multiplication effect between the two. In other words, the combined effects of neuroticism and stress are equivalent to the sum of their respective effects. In contrast, the interactive diathesis-stress model hypothesizes that there is a multiplication effect between neuroticism and stress (Mineka et al., 2020; Monroe & Simons, 1991). To the best of our knowledge, there have been no study to explore the combined effects of neuroticism factors by latent factor model and stress on anxiety symptoms. Understanding how different neuroticism factors and stress work together to predict anxiety symptoms will improve intervention plans and treatment strategies for the disease.

Above all, this study will investigate whether different neuroticism factors predict anxiety symptoms and whether different neuroticism factors and stress display reciprocal action on anxiety. This will be examined through longitudinal multiple evaluations in the adolescent and early adult samples, so as to identify the predisposing factors of anxiety in different ages, and so give relevant advice to the related mental health services.

## Materials & methods

### Subjects

In this study, 592 teenagers from a public middle School in Liuyang, Hunan, China were included in Group 1. Among them, 51.7% were male and 45.9% were female, with an age range from 14 to 19 years (average age: 16.32 years, standard deviation: 0.93).

In Group 2, 638 young adults (46.9% male and 52.4% female, aged 16–23 years) were included from Central South University, Hunan, China (average age: 20.11 years, standard deviation: 1.10).

All participants or their parents signed an informed consent form. The Ethics Committee of the School of Psychology, Capital Normal University approved the data collection of this study (Ethical Application No. CNUEC-2015-012).

### Data collection

For the adolescent samples, we first collected the initial assessment information from each participant. This baseline information was obtained through the self-assessment of the subjects through the completion of the following questionnaires: The NEO-FFIN Personality Inventory (NEO-FFIN) (Costa & McCrae, 1992); The adolescent life events questionnaire (ALEQ) (Hankin & Abramson, 2002) and The Multidimensional Anxiety Scale for Children (MASC) (March et al., 1997).

For the adult samples, a similar approach was adopted. The questionnaires included NEO-FFIN (Costa & McCrae, 1992); the General Social and Academic Hassles Scale (Blankstein, Flett & Koledin, 1991) and Anxious Arousal subscale in the Mood and Anxiety Symptoms Questionnaire (Clark & Watson, 1991).

Within 6 months following the collection of the baseline data, the subjects completed the stress and anxiety symptoms evaluation in the same place again every month. The descriptive statistics details of all the subjects were described in the Supplementary Files. The mean and SD for the initial assessment measures are presented in Table S1 and Table S2. Correlation analysis on different neuroticism factors and anxiety symptoms at the baseline time point was shown in Table S3.

### NEO-FFIN

NEO-FFIN (Costa & McCrae, 1992) is a questionnaire that works as a personality survey and aims to evaluate five personality features in the five-factor personality model. In the scale, 12 items are used to assess an individual’s neurotic personality. For the questions, a five-level scoring method is adopted. The lower the answer score, the lower level of disagreement there is with the question, while the higher the answer score, the higher level of agreement there is with the question. Yao verified the reliability of the NEO-FFIN questionnaire in China (Yao & Liang, 2010). For specific 12 items of the neurotic structure in NEO-FFIN, please refer to the section for the latent variable model and bi-factor model.

### ALEQ

ALEQ (Hankin & Abramson, 2002) was used to assess a common set of negative events among adolescents, covering studies, achievements, friendships, romantic relationships, and family relationships. In total the questionnaire included 57 items, e.g. “got a bad report card” in school activities, “Had a quarrel with a good friend” in friendship activities, “boyfriend/girlfriend broke up with you but you still want to go out with them” in love activities, and “your parents grounded you” in family activities. In order to reduce the degree of distortion caused by anxiety in the completion of the questionnaire, the ALEQs were scored according to the number of negative events that had occurred in the previous month for each subject. The higher the score, the more negative events the subject had experienced, and the more stress they had suffered. Previous studies have verified ALEQ as an effective and reliable method for the measurement of stress in Western adolescents (Calvete, Villardón & Estévez, 2008) and Chinese adolescents (Auerbach et al., 2007).

### MASC

MASC (March et al., 1997) consists of 39 self-reported items, and is designed to measure the severity of anxiety symptoms in the past week. Each item contains a statement that deals with anxiety (for example, “I feel restless or anxious,” “I worry about what others think of me,” etc.), with an answer score ranging from 0 (never applicable) to 3 (usually applicable). The scores range between 0 and 27 on this subscale. The higher the total score, the more severe the anxiety. The MASC has recently been recommended by a professional body as an effective tool for either the screening of anxiety in adolescents or for distinguishing anxiety from other disorders (Silverman & Ollendick, 2005). The high reliability and validity of the Chinese version of MASC as an anxiety measurement method has also been proven (Yao et al., 2007).

### SHS

The General Social and Academic Hassles Scale (SHS) (Blankstein, Flett & Koledin, 1991) is used to assess daily troubles related to family, work and study that have been experienced by students throughout the previous month. The scale consists of 30 items, and for each of which the score ranges from 0 to 6. The higher the SHS score, the higher the frequency of daily troubles over the past month. The Cronbach’s alpha coefficient for this scale is 0.91, which indicates a high internal consistency.

### MASQ

The Mood and Anxiety Symptoms Questionnaire (MASQ) (Clark & Watson, 1991) is a 62-item questionnaire for the assessing of symptoms of depression and anxiety over the previous week. In the MASQ Scale, MASQ anxiety arousal (MASQ-AA) is 17 items subscale which used to assess symptoms of anxiety, with the score for each item ranging from 1 to 5. The higher the MASQ-AA score, the more severe the anxiety. The Cronbach’s alpha coefficient for the Chinese version of the MASQ scale is 0.94, which indicates a high reliability and validity (Yang & Yao, 2009).

### Latent variable model and bi-factor model

In traditional personality trait assessments, the main methods include the sum-score approach and the subset sum-score (Hill et al., 2020). The main disadvantage for the sum-score approach is that a lot of valuable specific information may be concealed, while for the subset sum-score it is that special effects and common effects of different dimensions may be confused. Therefore, the latent variable model may be optimal, for it may capture valuable specific information from different dimensions, and thus have better predictive validity and overcome the shortcomings of the traditional personality trait assessment method to some extent.

Bi-factor model is one of the most common latent variable model (Bonifay, Lane & Reise, 2017; Rodriguez, Reise & Haviland, 2016). It uses general factors to explain the common effects of dimensions and several decomposed special factors to reflect the specificity of the dimensions. The bi-factor model assumes that there is no correlation between the general factors and the special factors. Thus, the bi-factor model may more directly interpret general factors and special factors than other latent variable model and traditional sum-score method in describing the multi-dimensional test structure (Chen et al., 2012).In this study, we use replicable item-cluster subcomponents of the NEO, which is based on Saucier (Chapman, 2007; Saucier, 1998), to conduct confirmatory factor analysis for bi-factor model. The confirmatory factor analysis was analyzed using Mplus (https://www.statmodel.com/index.shtml).

The replicable item-cluster subcomponents include self-reproach and negative emotion. Specifically, there are five items in the negative emotion: Worried: “I am not a worrier”; breakdown: “When I’m under a great deal of stress, sometimes I feel like I am going to pieces”; anxious: “I rarely feel fearful or anxious”; blue: “I rarely feel lonely or blue”; depressed: “I am seldom sad or depressed.” While in the self-reproach section, there are seven items: feels inferior: “I often feel inferior to others”; ashamed: “At times I have felt so ashamed that I just wanted to hide”; tense: “I often feel tense and jittery”; worthless: “Sometimes I feel completely worthless”; reactive: “I often get angry at the way people treat me”; helpless: “I often feel helpless and want someone else to solve my problems”; discourage: “Too often, when things go wrong, I get discouraged and feel like giving up.”

### Statistical analysis

Based on the previous studies, we utilized a multi-wave longitudinal model as the statistical method for the diathesis-stress model (Steca et al., 2014; Yang et al., 2010).Within this, the longitudinal data related to anxiety symptom scores were used to evaluate the predictive effects of the neuroticism factors on anxiety symptoms, the reciprocal effects of stress and neuroticism factors on anxiety symptoms, and whether neuroticism factors have a regulatory effect on the relationship between stress and anxiety symptoms or not. Specific calculations were completed by the SAS (version 9.0) (SAS Institute, 2015) mixed model program and the hierarchical linear model (HLM). A significant advantage of HLM in investigating growth is the flexibility of dealing with missing data, which can estimate by using all available data on the basis of maximum likelihood (Raudenbush & Bryk, 2002).The independent variables (predictors) are the scores for anxiety in baseline, different neuroticism factors and stress scores (ALEQ/SHS) of the subjects, as well as the scores for the interaction between different neuroticism factors and stress. The dependent variables are the anxiety scores of the subjects in MASC/MASQ. Based on our data, we found that the heterogeneous autoregressive structure was the most suitable covariance structures (ARH) for our analyses. Non-significant random effect parameters should be removed from the model before examining the fixed effect. In six multi-wave longitudinal analysis, all the random intercept, random slope, and the ARH parameter were significant and thus retained in the model. The covariance parameters for the ARH model were listed in Table S4 and Table S5.

## Results

### Bi-factor model

As shown in Table 1, the bi-factor model had good fit in both samples (the values of Chi-Square (WLSMV) and degree of freedom were minimum; the Comparative Fit Index and Tucker-Lewis Index were closest to 1, and RMSEA [90% CI] was close to 0). Therefore, the bi-factor model was appropriate for the measurement of neuroticism in this study.

**Table 1:** The model fit indices of the bi-factor model in the two groups.

	Adolescent	Early adult
Chi-Square	80.471	88.465
Degrees of Freedom	42	42
Comparative Fit Index	0.973	0.973
Tucker-Lewis Index	0.957	0.957
RMSEA [90% CI]	0.040 [0.026–0.053]	0.042 [0.030–0.054]

**Note:**

RMSEA: Root mean square error of approximation.

### General and special factors of neuroticism

**Figure 1:**
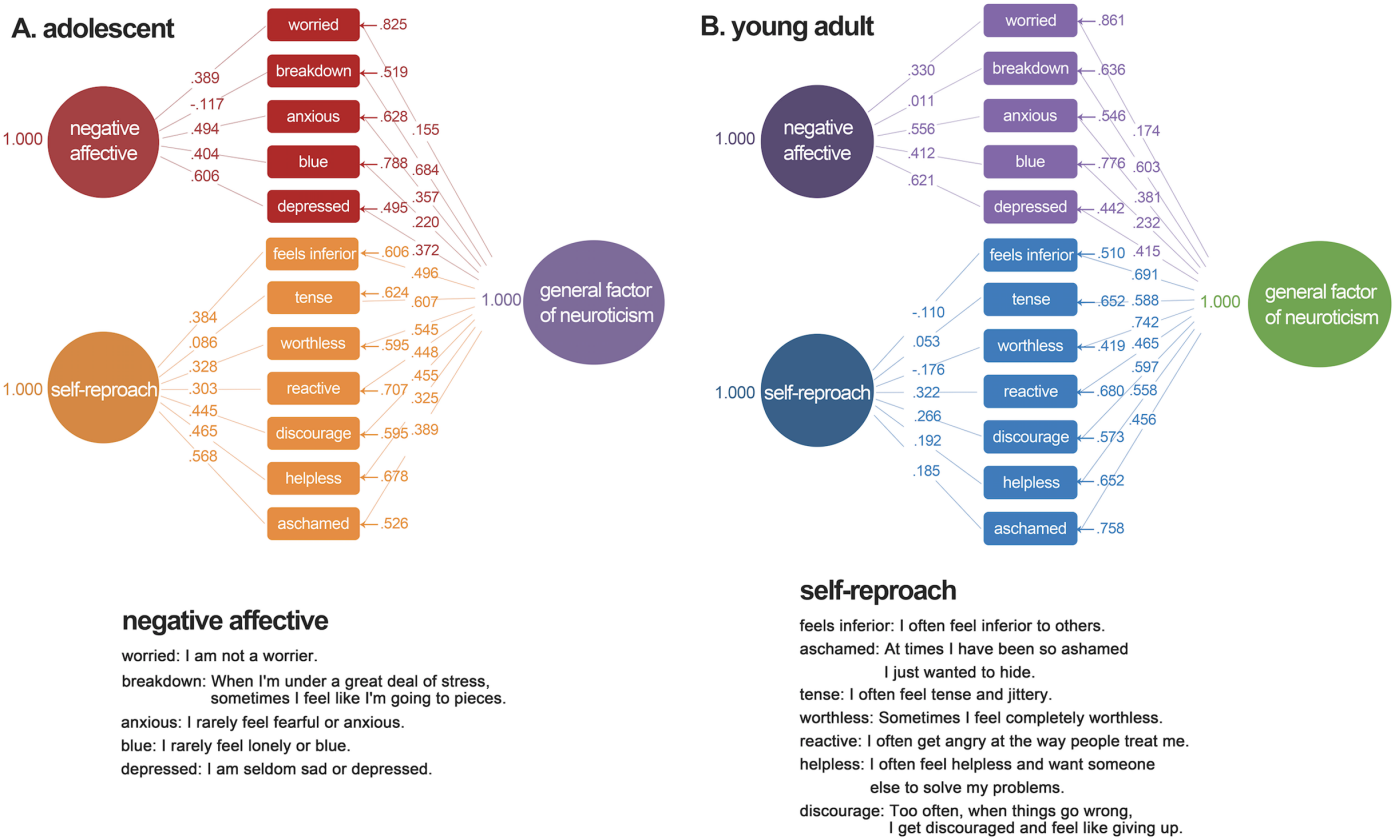
Standardized path coefficients for the bifactor model of neuroticism in the adolescent sample (A) and young adult sample (B).

Based on the bi-factor model, a general factor and two special factors (self-reproach factor and negative affective factor) were extracted from neuroticism. As presented in Table 2 and Table 3, in the bi-factor model, all the observed variables on the general factors are significant, indicating that all the items are good indicators of general factors. But in the sample of adolescent sample, the path coefficients of breakdown and tense on the corresponding special factors are not significant. In the sample of early adulthood, the path coefficients of breakdown, feels inferior, tense, and worthless on the corresponding special factors are insignificant. Most of the path coefficients on the general factors are greater than those on the domain specific factors. This insignificant result indicates that some domain specific factors are viable after the general factors are partialled out. The path coefficients for the bi-factor model are shown in Fig. 1.

**Table 2:** The standardized path coefficients of each parcel for bi-factor model in adolescent group.

	The general factor			Negative affectivity factor			Self- reproach factor		
Item	Estimate	Est./S.E.	*p*	Estimate	Est./S.E.	*p*	Estimate	Est./S.E.	*p*
worried	0.155	2.986	.003	0.389	7.636	<0.0001			
Breakdown	0.684	6.996	<0.0001	−0.117	−0.905	0.365			
Anxious	0.357	5.437	<0.0001	0.494	8.222	<0.0001			
Blue	0.220	4.247	<0.0001	0.404	8.060	<0.0001			
Depressed	0.372	4.855	<0.0001	0.606	9.046	<0.0001			
Feels inferior	0.496	7.599	<0.0001				0.384	4.711	<0.0001
Tense	0.607	6.741	<0.0001				0.086	0.514	0.607
Worthless	0.545	5.608	<0.0001				0.328	2.420	0.016
Reactive	0.448	7.980	<0.0001				0.303	4.094	<0.0001
Discourage	0.455	7.122	<0.0001				0.445	6.824	<0.0001
Helpless	0.325	4.162	<0.0001				0.465	6.838	<0.0001
Ashamed	0.389	4.273	<0.0001				0.568	7.363	<0.0001

**Note:**

All the items are referenced on the Neuroticism subscale of NEO five factor inventory.

**Table 3:** The standardized path coefficients of each parcel for bi-factor model in young adult group.

	The general factor			Negative affectivity factor			Self- reproach factor		
	Estimate	Est./S.E.	*p*	Estimate	Est./S.E.	*p*	Estimate	Est./S.E.	*p*
worried	0.174	4.082	<0.0001	0.330	6.883	<0.0001			
Breakdown	0.603	19.324	<0.0001	0.011	0.239	0.811			
Anxious	0.381	9.848	<0.0001	0.556	11.999	<0.0001			
Blue	0.232	5.384	<0.0001	0.412	8.960	<0.0001			
Depressed	0.415	10.908	<0.0001	0.621	12.980	<0.0001			
Feels inferior	0.691	23.014	<0.0001				−0.110	−1.049	0.294
Tense	0.588	19.131	<0.0001				0.053	0.780	0.435
Worthless	0.742	26.923	<0.0001				−0.176	−1.249	0.212
Reactive	0.465	9.352	<0.0001				0.322	2.847	0.004
Discourage	0.597	14.213	<0.0001				0.266	3.078	0.002
Helpless	0.558	14.691	<0.0001				0.192	2.025	0.043
Ashamed	0.456	10.829	<0.0001				0.185	1.665	0.096

**Note:**

All the items are referenced on the Neuroticism subscale of NEO five factor inventory.

### Predictive effect of the general factor of neuroticism on anxiety symptoms

Table 4 showed that the general factor of neuroticism at the baseline time point was a significant predictor for further anxiety symptoms (adolescents: F = 36.77, *p* < 0.0001, early adults: F = 30.44, *p* < 0.0001). Stress was also a significant predictor for anxiety symptoms (adolescents: F = 217.60, *p* < 0.0001; early adults: F = 10.74, *p* < 0.01). However, the interaction of general factor and stress only showed a predictive effect on anxiety symptoms in the early adulthood samples (adolescents: F = 0.13, *p* > 0.05; adults: F = 11.55, *p* < 0.01). The detail for the further moderate effect analysis was demonstrated in the Supplementary file.

**Table 4:** The general factor of neuroticism predicting within‐individual fluctuations in stress during the follow‐up interval.

Predictors		β	SE	F	df	*p*
Anxiety (baseline)	adolescent	10.02	0.60	276.69	566	<0.0001
	early adult	3.03	0.19	250.83	618	<0.0001
Stress	adolescent	6.92	0.47	217.60	2,688	<0.0001
	early adult	0.59	0.18	10.74	2,656	<0.01
General factor	adolescent	3.55	0.58	36.77	566	<0.0001
	early adult	1.03	0.19	30.44	618	<0.0001
Stress* General factor	adolescent	−0.16	0.45	0.13	2,688	=0.72
	early adult	0.61	0.18	11.55	2,656	<0.001

**Notes:**

General factor is a factor of neuroticism.

Neuroticism = Neuroticism subscale of NEO five factor inventory; Anxiety (adolescent) = The multidimensional anxiety scale for children (MASC); Anxiety (early adult) = The general social and academic hassles scale anxiety arousal subscale (MASQ-AA); Stress (adolescent) = The adolescent life events questionnaire (ALEQ); Stress (early adult) = The general social and academic hassles scale (MASC).

### Predictive effect of the reciprocal action of the negative affect factor and stress for anxiety symptoms

As shown in Table 5, in the relationship between the negative affective factors and anxiety, the negative affective factors of neuroticism were found to have a predictive effect on anxiety symptoms in adults (adolescents: F = 2.13, *p* > 0.05; early adults: F = 4.84, *p* < 0.05). However, we did not find the effect of reciprocal action of negative affective factors and stress on anxiety symptoms (adolescents: F = 0.65, *p* > 0.05; early adults: F = 0.31, *p* > 0.05).

**Table 5:** The negative affect factor of neuroticism predicting within‐individual fluctuations in stress during the follow‐up interval.

Predictors		β	SE	F	df	*p*
Anxiety (baseline)	adolescent	11.72	0.54	467.79	566	<0.0001
	early adult	3.32	0.19	322.06	618	<0.0001
Stress	adolescent	6.92	0.47	220.42	2,688	<0.0001
	early adult	0.61	0.18	10.89	2,656	<0.001
negative affect	adolescent	0.77	0.53	2.13	566	=0.14
	early adult	0.40	0.18	4.84	618	<0.05
Stress* negative affect	adolescent	0.36	0.45	0.65	2,688	=0.42
	early adult	0.10	0.18	0.31	2,656	=0.57

**Notes:**

Negative affect is a special factor of neuroticism.

Neuroticism = Neuroticism subscale of NEO five factor inventory; Anxiety (adolescent) = The multidimensional anxiety scale for children (MASC); Anxiety (early adult) = The general social and academic hassles scale anxiety arousal subscale (MASQ-AA); Stress (adolescent) = The adolescent life events questionnaire (ALEQ); Stress (early adult) = The general social and academic hassles scale (MASC).

### The predictive effect of the self-reproach factor for anxiety

As shown in Table 6, through a similar analysis, we found that the self-reproach factors of neuroticism had no predictive effects on anxiety symptoms (adolescents: F = 3.79, *p* > 0.05; early adults: F = 0.02, *p* > 0.05). In addition, we did not find any interaction effect of the self-reproach factors and stress on anxiety symptoms (adolescents: F = 0.74, *p*> 0.05; early adults: F = 0.64, *p*> 0.05).

**Table 6:** The self-reproach factor of neuroticism predicting within‐individual fluctuations in stress during the follow‐up interval.

Predictors		β	SE	F	df	*p*
Anxiety (baseline)	adolescent	11.57	0.56	431.13	566	<0.0001
	early adult	3.36	0.19	321.00	618	<0.0001
Stress	adolescent	6.97	0.47	218.84	2,688	<0.0001
	early adult	0.60	0.18	10.83	2,656	<0.001
Self-reproach	adolescent	1.04	0.53	3.79	566	=0.05
	early adult	−0.02	0.19	0.02	618	=0.90
Stress* Self-reproach	adolescent	−0.40	0.47	0.74	2,688	=0.39
	early adult	−0.14	0.18	0.64	2,656	=0.42

**Notes:**

Self-reproach is a special factor of neuroticism.

Neuroticism = Neuroticism subscale of NEO five factor inventory; Anxiety (adolescent) = The multidimensional anxiety scale for children (MASC); Anxiety (early adult) = The general social and academic hassles scale anxiety arousal subscale (MASQ-AA); Stress (adolescent) = The adolescent life events questionnaire (ALEQ); Stress (early adult) = The general social and academic hassles scale (MASC).

## Discussion

The bi-factor model achieved a satisfactory fit for the measurement of the neuroticism data in adolescence and early adulthood, and therefore, we believe that the bi-factor model is suitable for neuroticism evaluation. In addition, based on the multi-wave longitudinal model, our research supported previous relevant study results (Bienvenu & Stein, 2003; Vittengl, 2017), which showed that neuroticism (the general factor of neuroticism in our study) had a high predictive effect on anxiety symptoms, as well as a significant effect on anxiety symptoms in the diathesis (neuroticism) stress model. We found that the general factor of neuroticism independently predicted the occurrence of anxiety in samples of both adolescents and early adult college students. We also found it could interact with stress to influence anxiety symptoms in the adult samples, although their main effects are significantly larger than their interaction. However, we did not find self-reproach can predict anxiety symptoms independently, or self-reproach/negative emotional factors could influence anxiety symptoms when interacting with stress. Therefore, our result suggests that the general factor of neuroticism, but not the special factors can make an additive effect for anxiety symptom in face of stress, that means the homogeneity of neuroticism than heterogeneity can play a more significant role in further anxiety symptoms. In addition, the predictive effect of negative affect special factor on anxiety may not be shown until adulthood.

In our study, we did not find the interaction effect of neuroticism and stress on anxiety symptoms in adolescents, while in the samples of college students in early adulthood, we found that the interaction effect of neuroticism and stress was much lower than their respective direct effects. Therefore, we believe that both neuroticism and stress are significant independent predisposing factors for anxiety symptoms (He et al., 2018; Tackett & Lahey, 2017), neuroticism and stress may have additive effects on anxiety. In other word, we speculate that anxiety symptoms are most likely to develop among people who have high stress and neuroticism scores.

In addition, besides the general neuroticism factor, we also used the bi-factor model to extract two special factors from neuroticism, these being the self-reproach factor and the negative affective factor (Chapman, 2007; Saucier, 1998). The statistical analysis results showed that the main effect of negative affective factor on future depressive symptom was significant in the early adult samples. According to past studies, adults who exhibit more pain, anxiety, nervousness, or worry may be individuals who are more prone to have anxiety symptoms (Lahey, 2009; Tackett & Lahey, 2017). But we did not find such predictive effect in adolescents. We speculate that the role of negative affective factor may vary with age. Therefore, it is suggested that the negative affect factor should be considered as one of the predisposing factors for the determination of anxiety in adults, and when screening people at risk of anxiety we should pay more attention to those adults with high negative affect tendencies and neurotic personality.

Notably, we found that some items were not significant for the path coefficients of specific factors in bi-factor model, suggesting that these questions may have no significant effects on the corresponding specific factors. In addition, we found that this phenomenon was more pronounced in the adult group. We hypothesize that the reason may be that neurotic structures tend to be homogenous rather than heterogeneous with age. Past research has shown that neuroticism remains relatively stable after emerging in childhood (Barlow et al., 2014; Eaton, Krueger & Oltmanns, 2011). In addition, we chose the bi-factor model as a measure of neurotic structure mainly because it is a direct hierarchical model and assumes that higher-order factors and special factors are orthogonal (Beaujean, 2015; Lahey et al., 2021; Reise, 2012). Each item affects the general factors directly and sometimes influence a particular factor simultaneously. This attribute gives the bi-factor model an outstanding advantage in clearly explaining the formation mechanism of factors, especially for the general factors of neuroticism (Lahey et al., 2021; Reise, 2012). For other high-order models, they assume that high-order factors and low-order factors are statistically correlated, so it is difficult to explain the formation mechanism of high-order factors of neuroticism independently (Lahey et al., 2021; Mansolf & Reise, 2017).

This study has some limitations. First, in our samples, only Han nationality adolescents and young adult college students from Hunan in China were included, and so, there should be further studies to investigate whether these results can be generalized to other age groups, regions and races or not. Second, the data used in this study were mainly from the questionnaires completed through the subjects’ self-report. Although the reliability of these psychological survey scales has been proved, we cannot make any clinical diagnosis of mental disorders based on self-report questionnaires alone. Future studies should include more sophisticated diagnosis techniques for the assessment of anxiety, e.g., structured clinical interviews. Third, in this paper, we only discuss the influence of different factors of neuroticism and stress on anxiety symptoms. There are many other qualities that can affect anxiety symptoms, such as sleep quality, social support, etc (Jacobson, Lord & Newman, 2017; Xiong et al., 2019).Future research should explore more susceptibility factors for anxiety. Finally, the bi-factor model was well fitted and had a clear explanation for the general factors of neuroticism in this paper. However, our study did not use model comparisons to determine the best model for evaluating neurotic structures. Future studies will use a combination method of exploratory factor analysis and confirmatory factor analysis to assess the best statistical model for neuroticism.

## Conclusions

Our findings support the bi-factor model as an effective measurement method for neuroticism, as well as the applicability of the diathesis (neuroticism factors)-stress model in identifying the risk of anxiety. The study results have provided further insight into the relationship between different factors of neuroticism/stress and anxiety symptoms and highlighted the vulnerability of people with high the general factor of neuroticism when under stress. We found that a highly general factor and negative affect factor of neuroticism could predict anxiety in adults, and therefore may be able to contribute to the future identification of the risks involved in the emergence of anxiety symptoms. Furthermore, our exploration on the combined effect of neuroticism factors and stress on anxiety may provide some theoretical support for relevant institutions in the formulation of psychological health protection strategies for adolescents. In further studies, if the results of this study are able to be deeply integrated with clinical psychology and neuroscience methods, it may help us find more scientific behavioral indicators and biomarkers for anxiety symptoms as quickly as possible.

## Supplemental Information

10.7717/peerj.11379/supp-1 Supplemental Information 1 Supplemental Information

10.7717/peerj.11379/supp-2 Supplemental Information 2 Predicted slope of the relationship between stress and anxiety for individuals having either high or low levels of general factor of neuroticism scores.

10.7717/peerj.11379/supp-3 Supplemental Information 3 Means and standard deviations for all measures in adolescent sample.

10.7717/peerj.11379/supp-4 Supplemental Information 4 Means and standard deviations for all measures in early adult sample.

10.7717/peerj.11379/supp-5 Supplemental Information 5 Intercorrelations between Baseline measures.

10.7717/peerj.11379/supp-6 Supplemental Information 6 Estimates for covariance parameters of the ARH model in adolescent sample.

10.7717/peerj.11379/supp-7 Supplemental Information 7 Estimates for covariance parameters of the ARH model in early adult sample.

## Additional Information and Declarations

### Data Availability

The following information was supplied regarding data availability: Neuroticism factors data, the other measurements of the initial time, and all the code are available at GitHub: https://github.com/minnieho1115/Neuroticism-factors-and-anxiety-symptoms.
