# Supplementary material for: Neuroticism vulnerability factors of anxiety symptoms in adolescents and early adults: an analysis using the bi-factor model and multi-wave longitudinal model
Source: PeerJ. 2021 Jun 22;9:e11379. doi: 10.7717/peerj.11379 (PMC8231313; doi:10.7717/peerj.11379)
Supplement: Supplemental Information 1 [file peerj-09-11379-s001.docx]

**Descriptive statistics**

The mean and SD for the initial assessment measures are presented in Supplementary Table 1 and Supplementary Table 2**.** Correlation analysis on different neuroticism factors and anxiety symptoms at the baseline time point was shown in Supplementary Table 3. In the two samples, all of the three neuroticism factors had a significant positive correlation with anxiety, but the correlation between the general factor of neuroticism and anxiety symptoms was the highest.

Supplementary Table 1. Means and standard deviations for all measures in adolescent sample

Supplementary Table 2. Means and standard deviations for all measures in early adult sample

Supplementary Table 3.  Intercorrelations between Baseline measures.

**The covariance parameter for the autoregressive heterogeneous (ARH) model**

We examined the random-effects component of our model in order to decide whether the random-effects should be deleted from the model. Supplementary Table 4 and Supplementary Table 5 showed the estimates of covariance parameter for the final autoregressive heterogeneous (ARH) model.

Supplementary Table 4. Estimates for covariance parameters of the ARH model in early adult sample.

Supplementary Table 5. Estimates for covariance parameters of the ARH model in adolescent sample.

**Predictive effect of the general factor of neuroticism on anxiety symptoms**

To further compare the moderate effect of the general factor of neuroticism at different levels on stress and anxiety in young adult college students, the individuals with a general factor of neuroticism at high and low levels had their anxiety symptom scores calculated (1.5 SD) as experiencing stress at levels higher or lower than their average levels (1.5 SD). The trend was as shown in Supplementary Figure 1. In the figure, the slope of the line may be interpreted as the increase of anxiety symptom scores for every 1-point increase on the stress level. The SAS statistical analysis results showed that in the high scoring group for the general factor of neuroticism, the scores for anxiety symptoms increased with stress level, while in the low scoring group for the general factor of neuroticism, the scores for anxiety symptoms did not increase with stress level (the high score group: slope = 1.50, t = 4.70, p < 0.001; the low score group: slope = -0.32, t = -0.98, p > 0.05). Therefore, we believe that the high and low scoring groups for the general factor of neuroticism have significantly different response modes to anxiety symptoms under stress levels (slope = 1.82, t = 3.40, p < 0.01)(see in Supplementary Figure 1). Thus, in our opinion, the general factor of neuroticism may moderate the relationship between stress and anxiety symptoms in adults.

Supplementary Figure 1. Predicted slope of the relationship between stress and anxiety symptom for individuals having either high or low levels of general factor of neuroticism scores.
