# Supplementary material for: Neuroticism vulnerability factors of anxiety symptoms in adolescents and early adults: an analysis using the bi-factor model and multi-wave longitudinal model
Source: PeerJ. 2021 Jun 22;9:e11379. doi: 10.7717/peerj.11379 (PMC8231313; doi:10.7717/peerj.11379)
Supplement: Supplemental Information 3 [file peerj-09-11379-s003.docx]

Supplementary Table1. Means and standard deviations for all measures in adolescent sample

|  | Baseline | FU1 | FU2 | FU3 | FU4 | FU5 | FU6 |
| --- | --- | --- | --- | --- | --- | --- | --- |
| neuroticism | 33.40  (±7.93) |  |  |  |  |  |  |
| Stress | 115.45 | 103.42 | 99.36 | 96.7 | 93.98 | 93.49 | 91.99 |
|  | (±23.41) | (±22.46) | (±23.44) | (±26.15) | (±26.61) | (±25.41) | (±26.10) |
| Anxiety | 47.12 | 44.45 | 41.27 | 38.56 | 36.18 | 37.05 | 35.78 |
|  | (±15.90) | (±16.54) | (±17.66) | (±19.32) | (±20.29) | (±20.53) | (±21.00) |

Note.

Neuroticism = Neuroticism subscale of NEO Five Factor Inventory;

Stress (adolescent)= the Adolescent Life Events Questionnaire (ALEQ).

Anxiety (adolescent)= The Multidimensional Anxiety Scale for Children (MASC);

The mean (± standard deviation) is listed in the Table.
