# Supplementary material for: Neuroticism vulnerability factors of anxiety symptoms in adolescents and early adults: an analysis using the bi-factor model and multi-wave longitudinal model
Source: PeerJ. 2021 Jun 22;9:e11379. doi: 10.7717/peerj.11379 (PMC8231313; doi:10.7717/peerj.11379)
Supplement: Supplemental Information 4 [file peerj-09-11379-s004.docx]

Supplementary Table 2. Means and standard deviations for all measures in early adult sample

|  | | Baseline | | FU1 | | FU2 | FU3 | FU4 | FU5 | FU6 |
| --- | --- | --- | --- | --- | --- | --- | --- | --- | --- | --- |
| neuroticism | 30.86  (±7.12) | |  | |  | |  |  |  |  |
| Stress | 90.49 | | 76.39 | | 77.84 | | 74.36 | 69.90 | 66.86 | 68.00 |
|  | (±27.09) | | (±27.68) | | (±29.51) | | (±31.38) | (±31.95) | (±32.05) | (±32.86) |
| Anxiety | 21.75 | | 22.16 | | 22.61 | | 22.33 | 23.19 | 21.93 | 22.01 |
|  | (±5.33) | | (±6.09) | | (±7.06) | | (±6.81) | (±7.67) | (±6.85) | (±6.41) |

Note.

Neuroticism = Neuroticism subscale of NEO five factor inventory;

Anxiety (early adult) = The general social and academic hassles scale anxiety arousal subscale (MASQ-AA);

Stress (early adult) = The general social and academic hassles scale (SHS).

The mean (± standard deviation) is listed in the Table.
