# Supplementary material for: Neuroticism vulnerability factors of anxiety symptoms in adolescents and early adults: an analysis using the bi-factor model and multi-wave longitudinal model
Source: PeerJ. 2021 Jun 22;9:e11379. doi: 10.7717/peerj.11379 (PMC8231313; doi:10.7717/peerj.11379)
Supplement: Supplemental Information 5 [file peerj-09-11379-s005.docx]

Supplementary Table 3.  Intercorrelations between Baseline measures.

|  |  | Negative affect factor | Self-reproach  factor | The  General factor |
| --- | --- | --- | --- | --- |
| Stress | adolescent | .053 | .392** | .440*** |
|  | early adult | .033 | .010 | .204*** |
| Anxiety | adolescent | .139*** | .288*** | .526*** |
|  | early adult | .090* | .165** | .325*** |

Note. The negative Affect, self-reproach and general factor are factors of neuroticism

Neuroticism = Neuroticism subscale of NEO Five Factor Inventory;

Anxiety (adolescent)= The Multidimensional Anxiety Scale for Children (MASC);

Anxiety (early adult) = The General Social and Academic Hassles Scale anxiety arousal subscale (MASQ-AA);

Stress (adolescent)= the Adolescent Life Events Questionnaire (ALEQ).

Stress (early adult) = The General Social and Academic Hassles Scale (MASC);

*p<.05; **p<0.01; ***p < .001.
