# Supplementary material for: Neuroticism vulnerability factors of anxiety symptoms in adolescents and early adults: an analysis using the bi-factor model and multi-wave longitudinal model
Source: PeerJ. 2021 Jun 22;9:e11379. doi: 10.7717/peerj.11379 (PMC8231313; doi:10.7717/peerj.11379)
Supplement: Supplemental Information 6 [file peerj-09-11379-s006.docx]

Supplementary Table 4. Estimates for covariance parameters of the ARH model in adolescent sample.

| Factor | Covariance parameter | Estimate | SE | Z |
| --- | --- | --- | --- | --- |
| G | AHR | 0.43 | 0.04 | 11.58*** |
|  | random intercept | 103.40 | 10.82 | 9.56*** |
|  | random slope | 17.45 | 5.30 | 3.29*** |
| NA | AHR | 0.43 | 0.04 | 11.46*** |
|  | random intercept | 112.04 | 11.36 | 9.87*** |
|  | random slope | 17.42 | 5.31 | 3.28*** |
| SR | AHR | 0.43 | 0.04 | 11.53*** |
|  | random intercept | 111.81 | 11.28 | 9.92*** |
|  | random slope | 17.44 | 5.27 | 3.31*** |

Note.

G = general factor; NA = Negative affective factor; SR = self-reproach factor;

General factor, negative affective and self-reproach are factors of neuroticism.

Neuroticism = Neuroticism subscale of NEO five factor inventory.

* p <. 05; * * p <. 01;* * * p <.001
