# Supplementary material for: Neuroticism vulnerability factors of anxiety symptoms in adolescents and early adults: an analysis using the bi-factor model and multi-wave longitudinal model
Source: PeerJ. 2021 Jun 22;9:e11379. doi: 10.7717/peerj.11379 (PMC8231313; doi:10.7717/peerj.11379)
Supplement: Supplemental Information 7 [file peerj-09-11379-s007.docx]

Supplementary Table 4. Estimates for covariance parameters of the ARH model in early adult sample.

| Factor | Covariance parameter | Estimate | SE | Z |
| --- | --- | --- | --- | --- |
| G | ARH | 0.21 | 0.03 | 6.36*** |
|  | random intercept | 15.21 | 1.19 | 12.77*** |
|  | random slope | 4.35 | 1.16 | 3.77*** |
| NA | ARH | 0.22 | 0.03 | 6.42*** |
|  | random intercept | 15.91 | 1.24 | 12.84*** |
|  | random slope | 4.87 | 1.19 | 4.09*** |
| SR | ARH | 0.22 | 0.03 | 6.39*** |
|  | random intercept | 16.06 | 1.25 | 12.86*** |
|  | random slope | 4.80 | 1.19 | 4.02*** |

Note.

G = general factor; NA = Negative affective factor; SR = self-reproach factor;

General factor, negative affective and self-reproach are factors of neuroticism.

Neuroticism = Neuroticism subscale of NEO five factor inventory;

* p <. 05; * * p <. 01; * * * p <.001
